# Treatment of advanced, recurrent, resistant to previous treatments basal and squamous cell skin carcinomas with a synergistic formulation of interferons. Open, prospective study

**DOI:** 10.1186/1471-2407-9-262

**Published:** 2009-07-30

**Authors:** Lorenzo Anasagasti-Angulo, Yanelda Garcia-Vega, Silvia Barcelona-Perez, Pedro Lopez-Saura, Iraldo Bello-Rivero

**Affiliations:** 1 National Institute of Oncology and Radiobiology (INOR), Havana, Cuba; 2 Clinical Trial Department, Center for Genetic Engineering and Biotechnology (CIGB), Havana, Cuba

## Abstract

**Background:**

Aggressive non-melanoma skin cancer (deeply infiltrating, recurrent, and morphea form lesions) are therapeutically challenging because they require considerable tissue loss and may demand radical disfiguring surgery. Interferons (IFN) may provide a non-surgical approach to the management of these tumors. The aim of this work was to evaluate the effect of a formulation containing IFNs-α and -γ in synergistic proportions on patients with recurrent, advanced basal cell (BCC) or squamous cell skin carcinomas (SCSC).

**Methods:**

Patients with extensive, recurrent, resistant to other procedures BCC or SCSC received the IFN formulation peri- and intralesionally, three times per week for 3 weeks. They had been previously treated with surgery and/or radiotherapy or chemotherapy. Thirteen weeks after the end of treatment, the original lesion sites were examined for histological evidence of remaining tumor.

**Results:**

Sixteen elder (median 70 years-old) patients were included. They beared 12 BCC and 4 SCSC ranging from 1.5 to 12.5 cm in the longest dimension. At the end of treatment 47% CR (complete tumor elimination), 40% PR (>30% tumor reduction), and 13% stable disease were obtained. None of the patients relapsed during the treatment period. The median duration of the response was 38 months. Only one patient with complete response had relapsed until today. Principal adverse reactions were influenza-like symptoms well known to occur with interferon therapy, which were well tolerated.

**Conclusion:**

The peri- and intralesional combination of IFNs-α and -γ was safe and showed effect for the treatment of advanced, recurrent and resistant to previous treatments of BCC and SCSC in elder patients. This is the first report of such treatment in patients with advance non-melanoma skin cancer. The encouraging result justifies further confirmatory trials.

**Trial registration:**

Current Controlled Trials RPCEC00000052.

## Background

Non-melanoma skin cancers (NMSC) have a high incidence over the world, including basal cell carcinoma (BCC) and squamous cell skin carcinomas (SCSC), the most common neoplasms of the human being worldwide [1], irrespective of ethnicity [2]. The incidence of these tumors has risen [3], probably due to increase of aging population, improved detection, an increased use of tanning beds, and environmental factors such as increased sun exposure and ozone layer depletion which are known to increase the risk of BCC [4]. The role played by the immune system in immunocompetent patients with skin cancer is less clear, but ultraviolet (UV) radiation exposure is recognized as a critical factor in BCC pathogenesis, presumably partly because of resulting immune suppression [5]. P53 mutations have been shown in 30 to 50% of BCCs studied, and more than half of these mutations were UV-specific. More recently, BCC has bee associated with increase vascularity likely because of increased expression of CXCR-4, a receptor for stromal cell-derived factor 1 alpha (SDF-1) [6]. The Glioma-associated oncogene homolog (Gli) transcription factor family is greatly expressed in BCC lesions [7], where Hedgehog (Hh) signaling is required for growth of established BCCs [8]. Another way for development of NMSC is to avoid the immunesurvilance. BCCs have been shown to evade T cell response by secreting IL-10, by shedding ICAM-1 or by down-regulation of IFN-γ receptors (and thus HLA-class II antigens) [9] and by killing infiltrating citotoxic T cells [10].

Recurrence of BCC is not uncommon, approximately 12% with most treatment modalities. The rate of recurrence is positively correlated with tumor size and facial location. Up to 90% of recurrent cases occur on the head and neck. Aggressive histological BCC types are more prone to incomplete excision, recurrence, and metastasis. Tumors with aggressive histology tend to recur insidiously without early symptoms, leading to a delay in recognition that may compound the challenge of management. In a study of baso-squamous tumors, recurrence predictive factors included male gender, positive resection margins, and perineural or lymphatic invasion [11].

Well-known factors that have been shown to promote the development of SCSC include UV-radiation, immuno-suppression, exposure to ionizing radiation or chemical carcinogens, and infection with human papillomavirus (HPV). Malignant transformation of normal epidermal keratinocytes is the hallmark of SCSC. One critical pathogenic event is the development of apoptotic resistance through functional loss of TP53. Many other genetic abnormalities are believed to contribute to the pathogenesis of SCSC, including mutations of BCL2 and RAS. Likewise, alterations in intracellular signal transduction pathways, including epidermal growth factor receptor (EGFR) and cyclooxygenase (COX), have been shown to play a role in the development of SCSC [12].

Medical oncologists owing to success of local therapies rarely find SCSC. When advanced, SCSC requires systemic palliation, treatment with conventional chemotherapy, such as Cisplatin, is often precluded by patient's age or medical comorbidities [13].

The use of interferons (IFNs) in the treatment of BCC and SCSC has been reported [14-16], with a broad range of response (60% – 100%) and low recurrence rate (4%) [17]. IFN-α has been employed in the treatment of aggressive NMSC with 60% objective response (27% CR + 33% PR) [18] and 88.2% of complete response (CR) in invasive spinocellular carcinoma with tumor diameter lower than 2 cm [15].

However, previous report showed IFN as inefficacious in the treatment of aggressive BCCs, alone or in combination with retinoid to cure aggressive NMSC [18]. With high rates of tumor recurrence and with second primary tumors, patients with aggressive BCC and SCSC continue to have an unmet medical need, with devastating mortality, morbidity, and financial consequences [19]. The rising incidence, morbidity, and mortality of advanced BCC and SCSC are a major challenge for clinical oncologists. Promising agents with preclinical and early clinical results relevant to aggressive SCSC deserve a high priority.

Synergistic effects of combined treatment with IFN-α and -γ have been noted, suggesting that they operate on similar genes through different mechanisms. The cooperative induction of cytokine-specific transcription factors is one mechanism for producing reinforcing effects of distinct cell-surface ligands while still maintaining the specificities of the individual inducers [20]. The wide specter of biological activities shared by both IFNs could contribute to a more efficacious resolution of this difficult to treat skin tumors.

We have studied the application of a synergistic formulation of IFNs to explore, as primary objective, the safety and the clinical response with IFN combination treatment in advanced, recurrent and non-responder to previous treatments BCC and SCSC without any other therapeutic option. A secondary objective was the estimation of response duration.

## Methods

### Patients

Patients, both genders, older than 18 years, with advanced, recurrent, without response to previous treatments, not eligible for other available treatments, with histologically and clinically proven NMSC (BCC or SCSC), who gave their written informed consent to participate were enrolled. Tumors were every subtype, localization and size. The study excluded pregnant or nursing women, patients having at the entry to the study any additional topical therapy on or around the lesion or systemic chemotherapy, as well as patients with known hypersensitivity to IFN or with history of autoimmune disease. After being included, patients were permitted to incorporate concomitant onco-specific chemotherapy. The trial protocol was approved by the Ethics Committee and the Scientific Review Board of the "National Institute of Oncology and Radiobiology", Havana, in accordance with the ethical principles stated in the Declaration of Helsinki. All the patients were recluted and treated in the medical oncological cabinet (three times per week) suited in the "National Institute of Oncology and Radiobiology".

### Trial design

Was conducted a study for the treatment of cases as open prospective, non-controlled, in only one hospital. The stabilized pharmaceutical formulation of a synergistic combination of IFNs-α and -γ containing 3.0 MIU and sodium hydrogen-phosphate, dextran 40, sodium chloride, and human albumin (HeberPAG, Heber Biotec SA, Havana, Cuba) was employed for the treatment of patients.

This formulation was selected on the basis of in vitro anti-proliferative studies using isobologram analysis. During this study were identified the best combinations that lead to growth inhibition of several tumor cells lines, including basal cell carcinoma primary cultures from patients with BCC as well as established cells lines (Hep-2, Laringeal carcinoma, ATCC:CCL23), HepG2, Hepatoma, ATCC: HB-8065, and NCI-H125, Non-small cell lung cancer).

Each vial was reconstituted with 1 mL bacteriostatic water for injection and applied perilesional and/or intralesional three times per week for 3 weeks. The applications of IFN combination were practiced by medical doctor specialized in oncology with 15 years practical experience. For lesions > 4 cm, tumor area was measured, distributing it as surface of 1.5 cm^2 ^each. In areas, < 4 cm the product was injected in equidistant areas. The amount of product to be injected in 1 mL of total doses (10.0 MIU) per each 1.5 cm^2 ^of lesion surface was calculated. If the total amount to be injected was higher than 2 mL, the product was dissolved in appropriated volume in a sterile flask of approximately 10 mL. Overall, the procedures consumed approximately no more than 5 minutes.

The employed doses for IFN combination were defined as 3.0, 10.0, or 21.0 MIU based in preclinical and clinical data and taking in account that doses of IFN higher that 20 millions could produce sever adverse effects [21-23].

#### Chemotherapy indication

In cases where the tumor infiltrated bone or other cavities or was far from the needle to be injected, the intralesional synergistic combination of IFNs application was combined with its intramuscular application and combined with 1 cycle chemotherapy every 21 days (overall 4 cycles).

The doses and therapeutic schedule of chemotherapy followed the established therapeutic guidelines for these tumors. The chemotherapeutic doses were calculated following the Calver procedure [24]. The indicated cytostatic drugs were: Cisplatin: 50–100 mg/m^2^; Carboplatin: 100 mg/m^2^; Adriamycin: 50–70 mg/m^2^.

#### Surgery indication

Surgery were indicated as reconstructive option in one patient (non therapeutic surgery) and in case of no response.

Before treatment, each patient had a medical history, a physical and skin examination for disease assessment, documentation of concurrent medications, and laboratory tests (hemoglobin determination, platelets counts, white blood cell total, and differential counts). Patients were asked to avoid any other medication that could interfere with IFN action.

Subjects were examined as outpatients. At each visit during and after treatment, the investigators assessed the lesions and evaluated tissue conditions. Patients' follow-up examinations were done at weeks 0, 2, 4 and 16 after treatment onset. At week 16, laboratory tests were repeated. Lesion diameter (*d*) measurements were done using a Folding Magnifier, weekly and at week 16 and documented with photographs.

Thirteen weeks following therapy, the treatment sites were examined for clinical and/or histological evidence of remaining tumor. Patients were followed every 3 months for 1 one year after conclusion of treatment, and then 2 times a year until today.

### Clinical Evaluation

The study will explore if the application of IFN combination in so difficult to treat tumor is safe and provide benefit to the patients at designed doses.

All cases were examined as outpatients at regular intervals after injections. The main efficacy evaluation variable was the lesion response to treatment, based on the lesion area, 13 weeks after the end of treatment. It was categorized as complete response (CR): total disappearance of the tumor; partial response (PR): a clearly visible size reduction (>30%); stable disease (SD): < 30% reduction in the tumor size; and progression (P) defined as any increase in the lesion size. The CR responses were confirmed by computerized axial tomography in bone infiltration cases.

Long-term evaluation every 3 months for 1 year included clinical assessment for local relapses and tumor development at other locations. Response rates were based on the tumor area by measuring the diameter.

### Safety evaluation

Safety and tolerability were monitored by means of a rigorous adverse events control and their frequency calculated. During the treatment, patients were carefully monitored for side effects. Additionally, blood samples were taken for routine hematological and biochemical determinations.

### Laboratory procedures

Hematological counts and blood chemistry were done according to usual clinical laboratory procedures, using advanced automated analyzers. These included hemoglobin, hematocrit, leukocytes and platelets counts, transaminases, bilirubin, creatinine and urea. All laboratory analyses were done blindly.

### Statistic

Data were double entered and validated on Microsoft Access and then imported into SPSS version 13.0 for analysis. The frequency distributions for qualitative variables and central tendency and dispersion were estimated: mean, standard deviation, median, interquartilic range (QR), maximum and minimum values (range) for quantitative variables.

Effect variables

1. Response: (remission: total, partial, or not remission). Frequency distribution was estimated (IC 95%) using Bayesian statistic. The influence of demographic characteristic on clinical response were evaluated.

Safety variables:

2. Adverse events:

For each type of adverse event, were estimated frequency distribution (IC 95%) with classical and Bayesian statistic. Overall survival and duration of the response were based on the Kaplan-Meier method. The influence of demographic characteristics and appearance of adverse events were evaluated. The laboratory data were analyzed as a paired (initial – final result) using paired T student and Wilkinson tests, depending from Shapiro-Wilk test results.

## Results

### Patients demographic and baseline characteristics

The lesions included 12 BCC and 4 SCSC. The clinical forms were terebrant (9), nodular (3), mixed (2), and ulcerated (2). The dimension of the tested lesions was between 1.5 and 12.5 cm. Sites of tumors included face, neck and trunk.

The study was carried up between 2002 and 2006. Table 1 shows the demographic characteristics of 16 included patients. Patients were predominantly men (62.5%), between 31 and 89 years-old. All of them were white. There was not observed prevalence for smoking (37.5%) or alcohol consumption (12.5%). Sixth-eight percent of patients referred concurrent disease, predominantly arterial hypertension and cardiac insufficiency. All patients had one lesion. Most of recluted patients (93.8%) have been relapsed under treatments before entry this study, among them the most frequent were surgery (93.8%) and radiotherapy (81.2%). The time of tumor evolution was measured in months. The median of lesion evolution was 36 and 16 months for BCC and SCSC, respectively. The median of initial tumor area was 19.2 cm^2^. The main localization of tumor was in patient face (68.8%) and the terebrant clinical form was the most frequent (56.3%).

**Table 1:** Characteristics of the study population at entry

Variable N (16)		BCC	SSCC	Total
**Gender**	Male	7 (58.3%)	3 (75.0%)	10 (62.5%)
	Female	5 (41.7%)	1 (25.0%)	6 (37.5%)

**Skin color**	White			16 (100%)

	Mean ± SD	66.4 ± 14.1	72 ± 16.3	67.8 ± 14.3
**Age (Years)**	Median ± QR	70.5 ± 18.0	72.5 ± 31.0	70.5 ± 2.0
	Max; Min	(31, 81)	(54, 89)	(31, 89)

**Smoker**	Yes	6 (50.0%)	1 (25.0%)	7 (43.5%)
	No	6 (50.0%)	3 (75.0%)	9 (56.3%)

**Alcohol consumption**	Yes	1 (8.3%)	1 (25.0%)	2 (12.5%)
	No	11 (91.6%)	3 (75%)	14 (87.5%)

	No			4 (25%)
	Yes			12 (87.5%)
	
	**Diseases**		**Drugs for diseases control**	
	
			Captopril	1 (20%)
			Clortalidone	1 (20%)
			Captopril	1 (20%)
	AHT	5 (31.2%)	Hydrochlorothiazide	
			Captopril	1 (20%)
			Clortalidone	
			Enalaprile	1 (20%)
			Hydrochlorothiazide	
	
**Concurrent diseases (68% of patients)**	Cardiac insufficiency	4 (25%)	Dioxin	1 (25%)
	
	Bronchial asthma	2 (12.5%)	Prednisone	1 (50%)
	
	Diabetes	2 (12.5%)	Insulin	1 (50%)
	
	Lung ephysema	2 (12.5%)	Aminophyline	1 (50%)
	
	Allergic rhinitis	1 (6.2%)	Diphenhydramine	1 (100%)
	
	Breast tumor	1 (6.2%)		
			
	Duodenal ulcer	1 (6.2%)		
			
	Gastric ulcer	1 (6.2%)		
			
	Retinosis	1 (6.2%)		
			
	Cerebral ischemia	1 (6.2%)		

### Treatment compliment

Other specific drugs not established in the protocol for these patients were prohibited. In the study were indicated dipyrone or paracetamol associated to the difenhidramine for the relief of the adverse events. Six patients maintained during all the treatment time, the specific drugs for the control of the concurrent illnesses.

The 81.2% of patients completed the treatment schedule (9 IFN combination injections). Only three patients interrupted the treatment: one because of death, the other because the product did not penetrate the tumor tissue and spilled off, and the other by voluntary withdraw. All of them were included in clinical response analysis. Most patients received only IFN combination (75%), of them 31.3%, 25% and 43.7% received 3.0 MIU, 10.0 MUI, and 21.0 MIU, respectively. In four patients was necessary to include chemotherapy to the IFN combination treatment. The minimum total IFN-α doses received was 30 MIU and the maximum was 270 MIU. In the case of IFN-γ, the minimum total doses received were 4.5 MIU and the maximum 23 MIU. The most frequent used chemotherapy was Adriamycin plus Cisplatin. The total doses of Adriamycim employed were between 120 – 600 mg with a minimum of 2 cycles and maximum of 5 cycles. In the case of Cisplatin the total doses ranged between 195 and 850 mg for a minimum of 3 cycles and maximums 5 cycles.

### Clinical response

Fifteen patients were evaluated for clinical efficacy. Seven patients obtained CR (46.7%). Six patients had PR (40.0%). Only two patients were non-responder (13.3%). Eighty and 83.3% of patients with objective response (CR + PR) received 21 MIU and 10.0 MIU IFN combinations, respectively. In the group of patients that received 3.0 MIU of IFN combination all the patients obtained overall response (OR) (1 CR + 3 PR). Surgery was practiced in three patients after IFN combination treatment. One of them was a biopsy-exeresis that result in tissue fibrosis, the other was a surgery indicated as reconstructive option in one patient (non-therapeutic surgery) and the other in one case of no response.

It was done univariate analysis of demographic, base and control variables with respect to the occurrence of CR. In this case was not observed any significant influence of analyzed variables with respect to clinical response.

Only one out of 13 patients with clinical OR did not maintain sustained clinical response until 24 months. The median of sustained response was 38 months with a confidence interval of 95% (22.6 – 53.4). Five patients (3 SCSC and 2 BCC) dyed in the study (31.3%). The causes of the death were hepatic metastasis, pneumonia, and tumor progression. The mean survival was 42.3 months (95%, 29.4 – 55.2) (see Table 2). Most of the deaths were before 2 year.

**Table 2:** Mortality analysis using Bayes statistics

Variable		N (%)	IC 95% Bayes
Deaths	Yes	5 (31.3)	(15.5; 58.6)
	No	10 (62.5)	(41.0; 84.7)
	Unknown	1 (6.2)	

Causes of death	Hepatic breast carcinoma metastasis	1 (20.0)	(4.40; 64.5)
	Respiratory infection	2 (40.0)	(12.1; 77.6)
	Tumor progression	2 (40.0)	(12.1; 77.6)

Survival from the beginning of treatment	Mean	42.3 ± 6.6	(29.35; 55,21)
	Median	56.5 ± 29.4	(0.00; 114.2)

### Safety analysis

The exposition time to the IFN combination was between 3 and 18 weeks with a median doses of 166 MIU (30 – 270 MIU) IFN-α and 9 MIU IFN-γ (4.5 MIU – 23 MIU). Most of the patients (93.3%) developed adverse events. The most frequent were fever, chills and arthralgias (moderated) (see Table 3). The infrequent (rate = 1) adverse events were, dyspepsia, plexus brachial pain, otalgia, increase of aspartate aminotransferase (ASAT), sweating, nausea, local sepsis, abulia, dyspnea, cardiac arrhythmia, and ocular pain. Only one severe adverse event was reported and was related with previous received radiotherapy. Neither patient abandoned the treatment due to adverse event.

**Table 3:** Adverse events more frequently observed in patients treated with the synergistic IFN-α and -γ combination

Adverse events	Frequency	%
Fever	34	32.7

Chills	13	12.5

Arthralgias	11	10.6

Anorexia	10	9.6

Asthenia	7	6.7

Diarrhea	6	5.8

Myalgias	3	2.9

Chemosis	2	1.9

Erythema	2	1.9

Vomiting	2	1.9

Palpebral edema	2	1.9

## Discussion

The management of advanced and recurrent NMSC depends fundamentally from their aggressiveness, magnitude of infiltration, tumor size, and/or resistance to standard treatment, physical state of the patient, etc. The surgical resolution could be in some case an effective treatment; nevertheless, in most cases mutilating. The non-surgical treatment includes the radiotherapy, chemotherapy and immunotherapy. On the other hand, patients with several recurrences and/or resistance to other treatments cannot be tributaries of other therapies.

In the treated cases, BCC was more frequent than SCSC and located fundamentally in the face. The predominant clinical form was the terebrant, with median tumors area of 19.2 cm^2 ^of diameter and some with large extensions out of the skin [25,26]. The group of IFN combination treated patients was selected from those previously treated cases, in relapse after had received insufficient surgical excision, radiation therapy, or topic chemotherapy. The risk factors for the subclinic large extension of such tumors included tumor diameter over 2 cm, localized in the central part of the face or ears, long evolution time, incomplete eradication of the tumor, and perineural or perivascular infiltration. The extensive tumors (> 4 cm) or irregular edges associated more often with residual positive margins after surgical excision and have higher rate of recurrence than the smallest or well defined tumors [27].

Most patients suffered of concurrent cardiovascular or respiratory, or digestive illnesses, or diabetes, in concordance with epidemiological characteristics of Cuban population at these ages [28]. The concomitant treatment followed by patients apparently did not influence the results obtained with the IFN combination administration.

Only two patients did not respond to the treatment and they never received chemotherapy. One of them was a patient with sclerosant tumor to whom was impossible to administer the IFN combination. This patient dyed due to mammary carcinoma lung and liver metastasis, five years after the failed intent with IFN combination. The other patient without response suffered from an adenocystic basal carcinoma, who had received previously radiation therapy twice, four surgeries, of them two with reconstructions and long-term topic chemotherapy. In this case, the tumor was hardened, strongly adhered and with scars that interfered the drug diffusion. It is known that adenocystic basal carcinomas are more aggressive and resistant to radiotherapy. At this point was decided to carry out a reconstructive surgery. This patient was not included in the follow data. Only patients with objective clinical response were followed for relapses.

**Figure 1:**
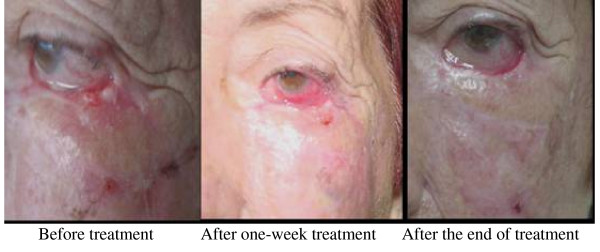
**Patient with BCC for more than 30 years treated several time with surgery and 5-FU and radiation therapies**. At the start IFN combination treatment the patient had a tumor lesion in free edge of his inferior reconstructed eyelid. After one-week treatment, it was observed in the place of tumor an ulcerated necrotic wound. At the end of IFN administration schedule a CR was achieved that prolonged for more than 7 years. Other BCC tumor around the treated lesion also improved.

**Figure 2:**
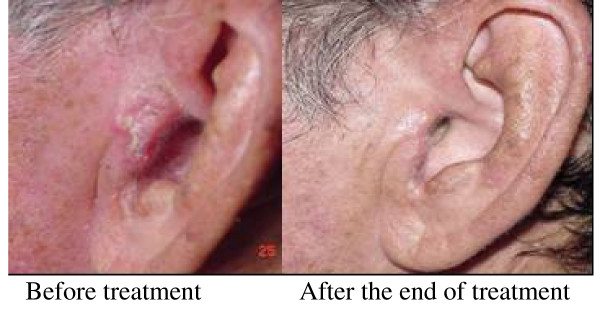
**Patients with BCC, treated previously with surgery and topic 5-FU with remaining lesion with cartilage infiltration and pain**. The patient had indicated another surgery for resection of the auricular pabellon plus reconstruction. Upon one week treatment with IFN combination pain disappeared and CR was achieved.

**Figure 3:**
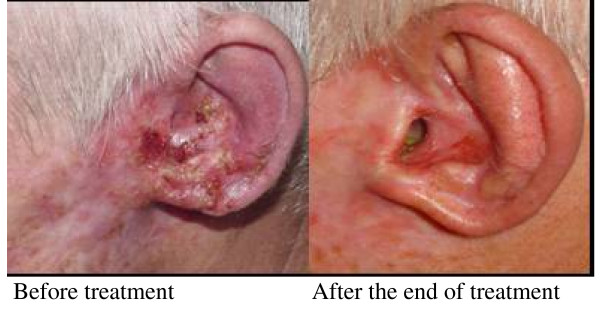
**Patients with SCSC grade I previously treated with surgery, topic 5-FU and radiotherapy**. He presented an extensive tumor lesion in the left ear and external auditory conduit and with indication of ear and external auditory conduit resection and reconstruction. After IFN combination administration a complete clinical response was observed that was sustained for 24 months.

**Figure 4:**
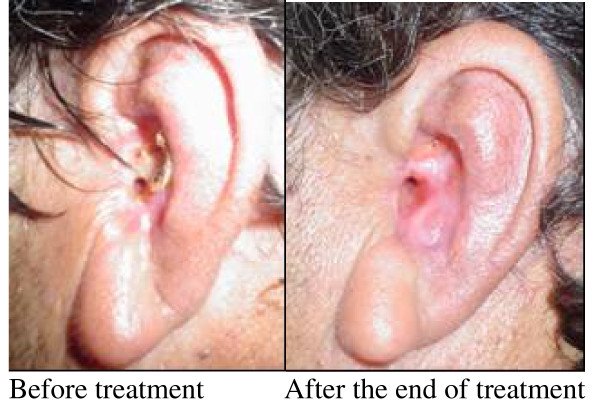
**Patient with BCC induced as consequence of radiotherapy of SCSC in the area of his left auricular pabellon for more than 10 years of evolution**. The patient had indicated to carry out partial temporalectomy including pabellon and conduit. He has been treated for three times with intralesional IFN combination, and obtained always partial or complete response but only for no more than 2 months of duration. The intralesional application was complemented with intramuscular administration that resulted in 85% of clinical response after 9 IFN applications.

With the IFN combination treatment was achieved an OR in 87% of cases (CR 47% + PR 40%). Most of 12 patients treated only with IFN combination (84%) obtained an OR, with completely disappearance of tumor in 50% of them. The responses in these so difficult to treat cases, even low or partial or the stabilization deserve the attention of international community because they are patients without other treatment options. In the Figures 1, 2, 3, and 4, are depicted some examples with partial and complete responses.

**Figure 5:**
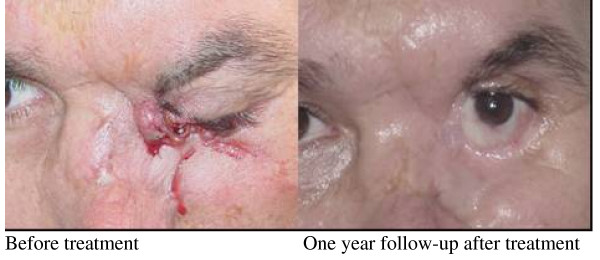
**Serious case of advanced BCC infiltrating and destruction of both eyelids in left eye, tear region, external wall of nostril and etmoids cells, constant exit of fetid liquid**. The therapeutic schedule for this patient consisted of intralesional administration of IFN combination with concomitant Cisplatin followed by intramuscular IFN combination. The patient achieved a CR for more than 3 years.

Patient treatment schedules had some variations due to differences between treated tumors, mainly in the spread to other zones or body structures and tumor size. The incorporation of chemotherapy [29] to the IFN combination administration showed impressive results in patients, with OR in 3 evaluated patients (2 CR + 1 PR), large tumors extended out of the skin and not reached by needle during the intratumoral IFN administration or with bone infiltration. In a patient with tumor orbital invasion the before proposed treatment option was the orbital exenteration, expanded or not [30,31]. The clinical result obtained for this patient is showed in figure 5 where is identifiable the organ preservation and functionality achieved after IFN combination application, verified by computerized axial tomography. Similar results are infrequent in the literature. Those reported were obtained in patients with less aggressive tumors, of lower size, without exhausting other therapeutic possibilities or that failed treatment one time either employing IFN-α along [32,33], combined with chemotherapy [34,35], or with other procedures [36,37], all of them with variable outcomes. With respect to the combination of IFNs-α and -γ has been reported studies in other types of tumors with variable responses; mainly with high dose of both IFNs [38-41].

Although the sample of patients that received chemotherapy combined with IFN combination is small, the results are impressive and very encouraging. The response is more prolonged and complete than the employment of the chemotherapy alone [45,46]. The combination of IFNs with chemotherapy has been reported to have synergistic effects by sensibilizing cells to apoptosis [42], acting as immunomodulator [43], potentiating anti-angiogenic effects [44] and others. This could explain the obtained and long lasting clinical response of patients treated with IFN combination plus chemotherapy.

The achieved results have a clear impact because convert the use of synergistic formulation of IFNs-α and -γ in a new potent antitumoral treatment to offer to this kind of patients with sustained long-term response, average of 38 months for the OR (22.6 – 53.4 months). Similar results have not been reported before employing either chemotherapy alone or combined with IFN-α and the obtained response has not exceeded 12 months average [45-47]. This is the first clinical results using this new stabilized formulation of synergistic combinations of IFN-α and IFN-γ (HeberPAG, HeberBiotec, Cuba).

It was reported high percentages of complete sustained response for five years, during the treatment of invasive recurrent non-melanoma skin cancer with chemotherapy of the cantus medium and orbit [29] but these cases were not of the magnitude of reported in our series of cases. Besides, the literature reports a high percentage of relapses before 12 months after treatment end. Additionally, the 50% of NMSC that recurred did during the two first years and 66% during three year, after being treated [48]. Patients, in whom the tumor disappeared after IFN combination treatment, had not yet recurred before 12 months. Even in the case that some one relapsed, it would be possible to repeat the treatment and to achieve a new period of diseases stabilization without arriving to major surgery.

Of five dyed patients; one dyed for causes not relate to treated tumor (metastasis of concomitant breast cancer); while in the remainder patients the causes of the death were related to treated tumor. The causes of death in 4 patients were as followed. Patient-1 dyed of serious hemorrhage in artery subclavia related to the tumor; patient-2 of bronchopneumonia by swallowed pus originating from the tumor tissue infection and perinasal exposed sinus and tumor cachexia, during tumor progression that destroyed face and part of the skull, finishing in patient prostration. Patient-3 death was caused by pneumonia related to ganglionar metastasis package of cheek and jaw spinocellular carcinoma that occupied supraclavicular grave, ulcerated toward the skin of anterior thorax and mediastine upper third. Patient-4 dyed because of tumor progression with cachexia and destruction of all centrofacial regions.

After combination treatment with IFNs, three patients were submitted for surgery. In one of them, upon achieving CR of more than 18 months (verified with biopsy), a reconstructive surgery was practiced in the lower eyelid of affected eye, to improve the ectropion and the functionality of the tear system (Figure 5). The second was to withdraw from the upper eyelid a small residual nodule of the extensive original lesion (small basal fibrous carcinoma) under disappearance by the replacement of tumor by the fibrous tissue. The third patient did surgery due to no clinical response with IFN combination applications.

The adverse events more frequent reported in the treated patients were fever (32.7%), chills (12.5%) and artralgia (10.6%) (see Table 3). Others represented less than 10% (anorexia, asthenia and diarrheas). The gastrointestinal related adverse events (anorexia, asthenia, diarrheas, vomiting, dyspepsia, queasiness) presented in this report were infrequent with regard to what reports the international literature for patients that received interferon (30 – 50%) [49].

Almost all adverse events reported were of low intensity for which was neither necessary to interrupt any treatment nor diminish the dose. Only one patient reported pain of serious intensity in the brachial plexus of affected side likely as consequence of radiation therapy received. This patient was maintained under IFN combination treatment with no future complication. This constitutes a very important fact because frequently due to IFN adverse events the treatment regimens are interrupted. This toxicity is increased when combines the chemotherpy with interferon [50,51], a phenomenon that never happened when combined the chemotherapy with the synergistic formulation of IFNs-α and -γ.

Almost, all the adverse events evolved in a favorable way during the treatment course, 51% even without the application of any other palliative therapy and 43% with the prescription of antipyretics. Only 4.6% of them (anorexia, asthenia and pain in the brachial plexus) persisted in two patients.

In this study the decrease of leukocytes count to 3 000 during the IFN combination treatment was observed one time in a single case. ASAT values were increased in a single occasion in two patients and never duplicated the values during the IFN combination treatment.

The adverse events reported in this study are similar to the described for the IFNs reported in the literature, as for type of adverse event, intensity and relation of causality, what indicates us that our synergistic formulation of IFNs possesses similar therapeutic safety profile as other market pharmaceutical presentation of IFNs.

The frequency of adverse events and its small magnitude, as well as the clinical effect of IFN combination, suggest that this new IFN formulation is harmless and sure, being able to be employed in similar therapeutic designs and prolonged treatment schedules, with the aims to offer to these patients an efficacious and safe therapeutic option.

Possible mechanisms involved in the observed clinical effects with the combination of IFNs-α and -γ could be the following. Intralesional IFN-α has been reported to initiate apoptosis of BCC cells via the CD-95 ligand-receptor interaction, a mechanism that could be reinforced by IFN-γ through the augmentation of CD-95 receptor [52]. The IFN-α, stimulating the expression of IFN-γ receptor (IFNGR1, and IFNGR2) [53] could contribute to reverse the observed low levels of this membrane receptor in BCC cells. Additionally it has been demonstrated that the in the presence of IFN-α, the intracellular signaling of IFN-γ is stronger [54]. Both IFNs have anti-angiogenic activities [55,56] and significantly repress CXCR4 gene expression that can lower the vascularity surrounded NMSC and impact in SDF-1-induced cell migration and proliferation of CXCR4-positive cells as have been demonstrated in head and neck squamous cell carcinomas for IFN-γ [57]. All these properties of IFNs-α and -γ and many others, likely contribute to enhanced anti-tumoral action of this new formulation of IFNs.

The prolonged observed clinical response may respond to the establishment of a more efficient and long-lasting eradication of tumors by a direct anti-tumor effect, and via activation of innate and adoptive immune responses coordinated and stimulated by both IFNs. This may comprise dendritic cells activation, tumor antigen presentation, and T-cell citotoxicity [58]. Such mechanism has been demonstrated for other treatments with administration of IFNs [59,60].

According to literature review, no one treatment for BCC is completely superior and other treatment methods do offer reliable alternatives. In this context, the treatment of NMSC with HeberPAG is a valuable option for many patients suffering from this skin cancer. Never before such kind of IFN formulation have been used for the treatment of patients and represents an encouraging therapeutic option for NMSC.

## Conclusion

The results obtained showed the safety and effectiveness of the synergic formulation of IFNs-α and -γ combination in the treatment of NMSC of any location, subtype and size, even in advanced state, and non-responder to previous therapies. The results of this study permitted to obtain unique data for IFN combination application in those non-melanoma skin tumors without any other therapeutic option or with the only option of a mutilating surgery. The results presented herein establish a candidate new therapeutic option for these difficult to treat NMSC. Further, more extensive, controlled clinical trials are encouraged to confirm this assessment.

## Abbreviations

BCC: Basal cell carcinoma; CR: Complete response; CIGB: Center for Genetic Engineering and Biotechnology; IFN: Interferon; MIU: Million international units; INOR: National Institute of Oncology and Radiobiology; OR: Overall response; PR: Partial response; P: Progression; SCSC: Squamous cell skin carcinomas;SD: Stable disease.

## Competing interests

Authors YGV, SBP PLS ands IBR are employees of the Center for Genetic Engineering and Biotechnology, Havana network, where the synergistic formulation of IFNs-α and -γ is produced. The rest of the authors have no competing interests at all.

## Authors' contributions

LAA conceived the study and was the main medical investigator. YGV conceived and designed the study, worked as study monitor, and took part in the results discussions and manuscript writing. SBP participated in data statistic analysis. PLS supervised the study and participated in results discussions. IBR took part in results discussions and wrote the manuscript. All authors read and approved the final manuscript.

## Pre-publication history

The pre-publication history for this paper can be accessed here:

http://www.biomedcentral.com/1471-2407/9/262/prepub
